# Longitudinal profile of antibody response to SARS-CoV-2 in patients with COVID-19 in a setting from Sub–Saharan Africa: A prospective longitudinal study

**DOI:** 10.1371/journal.pone.0263627

**Published:** 2022-03-23

**Authors:** Teklay Gebrecherkos, Yazezew Kebede Kiros, Feyissa Challa, Saro Abdella, Atsbeha Gebreegzabher, Dereje Leta, Abraham Desta, Ataklti Hailu, Geremew Tasew, Mahmud Abdulkader, Masresha Tessema, Getachew Tollera, Tsigereda Kifle, Zekarias Gessesse Arefaine, Henk HDF Schallig, Emily R. Adams, Britta C. Urban, Tobias F. Rinke de Wit, Dawit Wolday

**Affiliations:** 1 Mekelle University College of Health Sciences, Mekelle, Ethiopia; 2 Ethiopian Public Health Institute, Addis Ababa, Ethiopia; 3 Tigray Health Research Institute, Mekelle, Ethiopia; 4 Department of Medical Microbiology, and Infection Prevention, Experimental Parasitology Unit, Amsterdam Institute for Infection and Immunity, Academic Medical Center at the University of Amsterdam, Amsterdam, The Netherlands; 5 Liverpool School of Tropical Medicine, Liverpool, United Kingdom; 6 Amsterdam Institute Global Health and Development, Academic Medical Center, University of Amsterdam, Amsterdam, The Netherlands; Qatar University, QATAR

## Abstract

**Background:**

Serological testing for SARS-CoV-2 plays an important role for epidemiological studies, in aiding the diagnosis of COVID-19, and assess vaccine responses. Little is known on dynamics of SARS-CoV-2 serology in African settings. Here, we aimed to characterize the longitudinal antibody response profile to SARS-CoV-2 in Ethiopia.

**Methods:**

In this prospective study, a total of 102 PCR-confirmed COVID-19 patients were enrolled. We obtained 802 plasma samples collected serially. SARS-CoV-2 antibodies were determined using four lateral flow immune-assays (LFIAs), and an electrochemiluminescent immunoassay. We determined longitudinal antibody response to SARS-CoV-2 as well as seroconversion dynamics.

**Results:**

Serological positivity rate ranged between 12%-91%, depending on timing after symptom onset. There was no difference in positivity rate between severe and non-severe COVID-19 cases. The specificity ranged between 90%-97%. Agreement between different assays ranged between 84%-92%. The estimated positive predictive value (PPV) for IgM or IgG in a scenario with seroprevalence at 5% varies from 33% to 58%. Nonetheless, when the population seroprevalence increases to 25% and 50%, there is a corresponding increases in the estimated PPVs. The estimated negative-predictive value (NPV) in a low seroprevalence scenario (5%) is high (>99%). However, the estimated NPV in a high seroprevalence scenario (50%) for IgM or IgG is reduced significantly to 80% to 85%. Overall, 28/102 (27.5%) seroconverted by one or more assays tested, within a median time of 11 (IQR: 9–15) days post symptom onset. The median seroconversion time among symptomatic cases tended to be shorter when compared to asymptomatic patients [9 (IQR: 6–11) vs. 15 (IQR: 13–21) days; p = 0.002]. Overall, seroconversion reached 100% 5.5 weeks after the onset of symptoms. Notably, of the remaining 74 COVID-19 patients included in the cohort, 64 (62.8%) were positive for antibody at the time of enrollment, and 10 (9.8%) patients failed to mount a detectable antibody response by any of the assays tested during follow-up.

**Conclusions:**

Longitudinal assessment of antibody response in African COVID-19 patients revealed heterogeneous responses. This underscores the need for a comprehensive evaluation of seroassays before implementation. Factors associated with failure to seroconvert needs further research.

## Introduction

A new coronavirus, severe acute respiratory syndrome coronavirus 2 (SARS-CoV-2), has recently emerged leading to a global pandemic [1]. Although molecular tests remain the mainstay diagnostic tool for identifying SARS-CoV-2 infected patients [2, 3], their use is limited due to multitude of factors. Molecular tests require complex laboratories and highly trained expertise, creating a challenge for their use in resource–constrained settings. In addition, the sensitivity of molecular assays for SARS-CoV-2 is variable, depending on sample type, disease severity and sampling time after onset of infection [3]. To overcome some of these obstacles, serological tests, including rapid diagnostic tests (RDTs) for use at Point-of-Care (PoC), have emerged as an important alternative. Serological testing for SARS-CoV-2 plays an important role for epidemiological studies and in aiding the diagnosis of (previous) COVID-19 in PCR–negative patients [4–7]. In addition, serology testing may be important in the assessment of convalescent plasma, and also in the identification, and quantification of vaccine responses [8, 9]. Several reports have shown that median seroconversion time is 7–14 days after symptom onset [7, 10–12]. In addition, it has been demonstrated that antibody levels may correlate with COVID-19 severity [11, 12].

Most of the available data on the longitudinal profile of antibody responses are from high income countries (HICs), while little is known from low and medium income countries (LMICs), in particular from Sub–Saharan Africa (SSA) [13, 14]. In addition, applied SARS-CoV-2 serological tests evaluated in HICs might experience significantly higher cross-reactivity in SSA that may impact on the specificity of the assays, as background infectious diseases are entirely different [15, 16]. Therefore, there is a need for quality assessment of antibody responses to SARS-CoV-2 in the context of SSA. Such tests are needed urgently to scale–up SARS-CoV-2 surveillance, and diagnosis in LMICs, including in SSA.

In this study, we aimed to determine the longitudinal antibody response of SARS-CoV-2 using different assays in SSA context. In addition, we assessed seroconversion dynamics, and characteristics of different anti-SARS-CoV-2 antibodies. To the best of our knowledge, this study is the first prospective study to evaluate the kinetics of anti–SARS-CoV-2 antibody response, as well as seroconversion patterns among COVID-19 patients in SSA.

## Materials and methods

### Study population

The study is part of the ongoing Profile-Cov study, a prospective observational cohort study in Ethiopia with rapid diagnostic profiling of SARS-CoV-2 in the context of persistent immune activation (Clinicaltrials.gov: NCT04473365). Consecutive patients with confirmed real–time polymerase chain reaction (RT-PCR) test result were enrolled. They were recruited from Mekelle University College of Health Sciences (Kuyha COVID-19 Isolation and Treatment Center), Mekelle City, Northern Ethiopia, in a prospective fashion between July 15 and October 28, 2020. Individuals presenting to the Isolation and Treatment Center were screened for SARS-CoV-2 infection with a naso-pharyngeal swab and a RT-PCR. Following the declaration by the WHO that COVID-19 has become pandemic, the Ethiopian Ministry of Health implemented a mass screening of all travelers, people who had come in contact with COVID-19 patients, those from high-risk settings (e.g. health-care workers), as well as those with symptoms and signs suggestive of SARS-CoV-2 infection [17]. All cases with a confirmed SARS-CoV-2 infection were admitted to dedicated COVID-19 Isolation and Treatment Centers. Patients were admitted irrespective of clinical severity status. Whenever patients progress to severe or critical COVID-19, they were admitted to the intensive care unit (ICU).

Sociodemographic, clinical data and laboratory data were collected using standardized Case Record Forms (CRFs) adapted from the International Severe Acute Respiratory and Emerging Infection Consortium’s (ISARIC) CRFs for emerging severe acute respiratory infections [18]. Patient’s clinical status was stratified following WHO criteria as asymptomatic, mild/moderate, severe (with dyspnea, respiratory rate ≥ 30 breaths per minute, O_2_ saturation ≤ 93%, lung infiltrates ≥ 50% of the lung fields within 24–48 hours), and critical (with respiratory failure, septic shock, and/or multiple organ failure) [19]. For this study, asymptomatic and mild/moderate cases were considered as non–severe cases and both severe and critical were considered as severe cases. All data were then entered onto electronic medical records.

For analysis of kinetics profile samples were drawn serially from SARS-CoV-2 RT-PCR confirmed patients. Overall, patients were followed for a median of 31 [interquartile range (IQR): 16–32] days, and ranging between 5 to 39 days. Whenever possible, samples were drawn every 3 days during follow-up. Thus, multiple samples were drawn per individual at a given time interval (0–3, 4–6, 7–9, 10–12, 13–15, 16–18, 19–21, 22–24, 25–27, 28–30, 31–33, 34–36, 37–39, and ≥ 40 days after onset of symptoms). Notably, only one sample per time interval was included from a single participant. Specimens were immediately transported to the Central Laboratory of the university hospital, and separated plasma was frozen to –80°C until further analysis.

For determining specificity, RT-PCR negative specimens (n = 100) were obtained during COVID-19 pandemic (August and September 2020) from individuals not suspected of SARS-CoV-2infection. In addition, pre–pandemic specimens (n = 50) collected in 2017 from patients with other infections were included as negative controls in the analysis of specificity.

### Sample size

The sample size required to assess kinetics of antibody response to SARS-CoV-2 using serology assay, based on an expected sensitivity (and specificity) of 80% and a desired error margin of ±10% and an alpha level of 5%, was estimated to be 62 COVID-19 cases and 62 non COVID-19 controls. To take into account prevalence variability (e.g. 80%), we aimed to recruit a minimum of 150 (75 per group) individuals.

### Laboratory assays

SARS-CoV-2infection was confirmed by RT-PCR on samples obtained from nasopharyngeal swabs, as described previously [17]. The swabs collected were put in virus transport media and sent immediately to the central laboratory. RNA was extracted using the MagNA Pure 96 (Roche, Germany). RT-PCR for the detection of SARS-CoV-2infection was based on the LightMix® Modular SARS and Wuhan CoV E–gene kit (TIB Molbiol, Berlin, Germany). Briefly, a 25 μL reaction contains 5 μL of RNA, 12.5 μL of 2 × reaction buffer provided with the Superscript III one step RT-PCR system with Platinum Taq Polymerase (LightCycler Multiplex RNA Virus Master (Roche) containing 0.4 mM of each deoxyribonucleotide triphosphates (dNTP) and 3.2 mM magnesium sulphate), 1 μL of reverse transcriptase/Taq mixture from the kit, 0.4 μL of a 50 mM magnesium sulphate solution (TaqMan Fast Virus 1–Step Master Mix (Thermo Fisher)), and 1 μg of nonacetylated bovine serum albumin (Roche). Thermal cycling was performed at 55°C for 10 min for reverse transcription, followed by 95°C for 3 min and then 45 cycles of 95°C for 15s, 58°C for 30s. We used the thermocycler from Applied Biosystems ViiA7 thermocycler (Applied Biosystems, Hong Kong, China).

Five different commercial kits were studied (**S1 Table**). These included four lateral flow immune assays (LFIAs), i.e. Canea, Cellex, Innovita, and Vivachek that target the nuclear (N) and spike (S) proteins of SARS-CoV-2, and the Roche elecsys electrochemiluminescent immunoassay (ECLIA) that targets the nucleocapsid protein (NP). Whereas the LFIAs are designed to detect IgM and IgG against SARS-CoV-2, the Roche elecsys assay detects anti-SARS-CoV-2 total antibody levels. The assays were selected based on their availability in the local market as well as performance data. Testing was undertaken following manufacturer’s instructions for each assay. For the LFIAs, testing involved the addition of 10 μL of plasma to the sample well, and 80–100 μL of buffer to an adjacent well, followed by 15 minutes incubation at room temperature. The result was based on the appearance of colored bands, interpreted as positive when control and test bands were visible, as negative when only the control band was visible, or as invalid when the control band was not visible. All results were read by two technologists independently and photographed in real time. In the event of a discrepant report, reading by a third technologist served as a tie breaker. The Roche elecsys ECLIA was performed using the Roche Cobas® e411 immunoassay analyzer. Anti-SARS-CoV-2 total antibody levels were expressed as cut-off index (COI), and a value of ≥ 1.0 was considered as a positive result as per the manufacturer’s instructions. The individuals running the serology assays were blinded as to the origin of the sample, clinical data of the study participants, or to the reference SARS-CoV-2 results.

Seroconversion was ascertained if a patient with a negative antibody test at time of enrollment becomes positive with any of the assays tested during follow-up. For estimating the day of seroconversion, the days post symptom onset were determined for symptomatic cases. For asymptomatic patients, we added 6 days (median time between symptom onset and date of positive PCR testing among symptomatic cases) to the PCR testing date.

### Statistical analysis

Baseline characteristics for continuous variables were expressed as the median with IQR, and for categorical variables as proportions. Whereas categorical variables were compared using Fisher’s exact test or χ² test, continuous variables were compared by Mann–Whitney U or Kruskal–Wallis tests as appropriate. The primary outcome for this study was the proportion of positivity rates (sensitivity) of the various serology tests, stratified by Ig isotypes (IgM, or IgG, or total Ig), and according to sampling time (days after symptom onset) among specimens derived from RT-PCR confirmed COVID-19 cases. Specificity was determined among RT-PCR negative, or pre-COVID specimens. Positivity and specificity rates were presented as proportion and 95% confidence interval (CI) limits. Secondary analysis included performance characteristics of the assays stratified by the patient’s clinical severity status. Positive predictive values (PPV) and negative predictive values (NPV) were determined taking into consideration a population prevalence of 5%, 25%, and 50% SARS-CoV-2 infection. Agreement between different assays was determined by Kappa statistics. P values <0.05 were considered statistically significant. Reporting of the diagnostic performance of the assays in this study was according to the STARD (Standards for Reporting of Diagnostic Accuracy Studies) guidelines. Data were analyzed using STATA (Statistical package v. 14.0, StataCorp, Texas, USA).

### Ethical considerations

Participants provided written informed consent to participate in the Profile-Cov study. The study protocol was reviewed and approved by the Health Research Ethics Review Committee (HRERC) of Mekelle University College of Health Sciences (#ERC 1769/2020). Written informed consent was obtained from all study participants. However, specimens used as controls from pre–pandemic period were specimens from biobank and individual study participant consent was not obtained. Nonetheless, all personal identifiers were de–linked from the original sources, and the HRERC has reviewed and approved it.

## Results

### Characteristics of study participants

We initially enrolled a total of 120 patients. However, 18 were lost to follow–up, the majority were lost after their SARS-CoV-2 PCR test results, and few had only ≤ 1 sample, and we were not able to draw sufficient longitudinal serial specimens. Thus a total of 102 patients were eventually included, and detailed clinical characteristics of the enrolled participants is provided in **Table 1**. The majority were male (76.5%), with a median age of 33 (IQR: 27–42) years old, and 12.8% were aged ≥ 60 years. There was no difference in age between male and female study participants. Whereas 32/102 (31.4%) were asymptomatic, the remaining 70/102 (68.6%) were symptomatic (36 had mild/moderate and 34 had severe/critical clinical presentation). COVID-19 patients presenting with severe clinical status were older [median age 50 years (IQR: 38–66) vs. 29 years (IQR: 26–37); p<0.00001]. In addition, severe COVID-19 patients manifested with a significantly higher frequency of symptoms when compared to those with non-severe COVID-19. Patients with severe disease symptoms when compared to non-severe cases were more febrile, dyspneic, had more cough, chest pain, and head ache. The frequency of sore throat, nasal congestion, loss of smell and/or taste, diarrhea and myalgia were not different between both patient groups. Patients with severe COVID-19 were significantly more likely to be tachypneic and tachycardic when compared to those with non-severe disease. In addition, lymphocyte and hematocrit counts were significantly lower among severe COVID-19 patients compared to those with non-severe clinical manifestations. Comorbidities, including non-communicable diseases, were significantly higher among severe COVID-19 cases when compared to the non-severe. In addition, all severe cases of COVID-19 patients were admitted to the intensive care unit, received supplemental oxygen therapy, or mechanical ventilation. Finally, mortality was significantly higher among severe cases of COVID-19 patients.

**Table 1:** Baseline clinical characteristics of study participants.

Characteristic	All patients N = 102	Non-severe patients (asymptomatic and mild/moderate) N = 68	Severe patients (severe and critical) N = 34	P value
**Socio-demographic features:**				
Gender (male)	78 (76.5%)	52 (76.5%)	26 (76.5%)	1.000
Age in years [median (IQR)]	33 (27–42)	29 (26–37)	50 (38–66)	<0.00001
Age group [years]				
< 60	89 (87.3%)	68 (100.0%)	21 (0.00%)	<0.0001
≥ 60	13 (12.8%)	0 (0.0%)	13 (38.2%)	
**Clinical symptoms and signs:**				
Fever	29 (28.4%)	9 (13.2%)	20 (58.8%)	<0.0001
Dyspnea	38 (37.3%)	8 (11.8%)	30 (88.2%)	<0.0001
Cough	48 (47.1%)	19 (27.9%)	29 (85.3%)	<0.0001
Chest pain	9 (8.8%)	2 (2.9%)	7 (20.6%)	0.003
Sore throat	10 (9.8%)	5 (7.4%)	5 (14.7%)	0.239
Headache	27 (26.5%)	12 (17.7%)	15 (44.1%)	0.004
Nasal congestion	8 (7.8%)	7 (10.3%)	1 (2.9%)	0.193
Loss of smell and/or taste	4 (3.9%)	3 (4.4%)	1 (2.9%)	0.718
Diarrhea	1 (1.0%)	0 (0.0%)	1 (2.9%)	0.155
Myalgia	13 (12.8%)	7 (10.3%)	6 (17.7%)	0.294
Temperature (≥ 37.3°C)	5 (4.9%)	0 (0.0%)	5 (14.7%)	0.001
Systolic blood pressure (median mmHg, IQR)	112 (105–121)	112 (102–120)	122 (110–131)	0.010
Diastolic blood pressure (median mmHg, IQR)	74 (68–80)	73 (68–79)	77 (70–83)	0.110
Respiratory rate (median breaths/minute, IQR)	22 (19–28)	20 (18–22)	29 (28–31)	<0.0001
Heart rate (median beats/minute, IQR)	82 (76–98)	78 (68–83)	105 (98–112)	<0.0001
**Laboratory markers:**				
Lymphocyte, x10^9^/L	1.6 (1.1–1.9)	1.7 (1.6–2.0)	1.4 (0.9–1.7)	0.029
Hematocrit, %	42.9 (37.5–46.1)	46.1 (43.0–47.4)	42.0 (31.0–45.3)	0.016
Platelet count, x10^9^ /L	234 (174–319)	236 (187–266)	234 (174–353)	0.650
Alanine aminotransferase concentration, U/L	48 (33–62)	51 (49–75)	41 (30–50)	0.109
Creatinine concentration, mg/dL	0.6 (0.4–0.8)	0.5 (0.2–0.6)	0.7 (0.4–0.9)	0.087
**Comorbidities:**				
Comorbidity (at least 1)	25 (24.5%)	2 (2.9%)	23 (67.7%)	<0.0001
Non–communicable diseases	19 (18.6%)	2 (2.9%)	17 (50.0%)	<0.0001
**Outcomes:**				
Admission to ICU	9 (8.8%)	0 (0.0%)	9 (26.5%)	<0.0001
Supplemental oxygen	34 (33.3%)	0 (0.0%)	34 (100.0%)	<0.0001
Invasive mechanical ventilation	5 (4.9%)	0 (0.0%)	5 (14.7%)	0.001
Death	5 (4.9%)	0 (0.0%)	5 (14.7%)	0.001

Abbreviations: ICU: intensive care unit; IQR: interquartile ranges

### Longitudinal antibody response to SARS-CoV-2 and performance of serological assays

**Fig 1:**
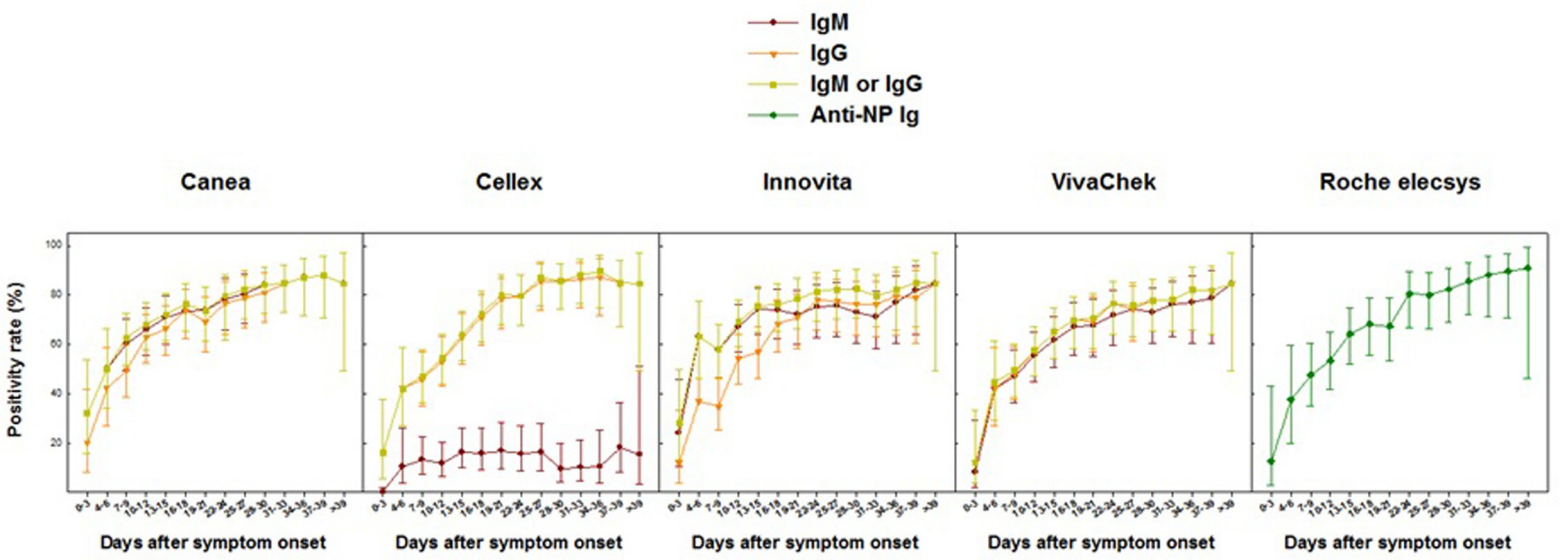
Kinetics of positivity rate (95%CI) of IgM, IgG, IgM or IgG LFIAs anti-NP total Ig at different time points after symptom onset. Raw data included as S2 Table.

**Fig 2:**
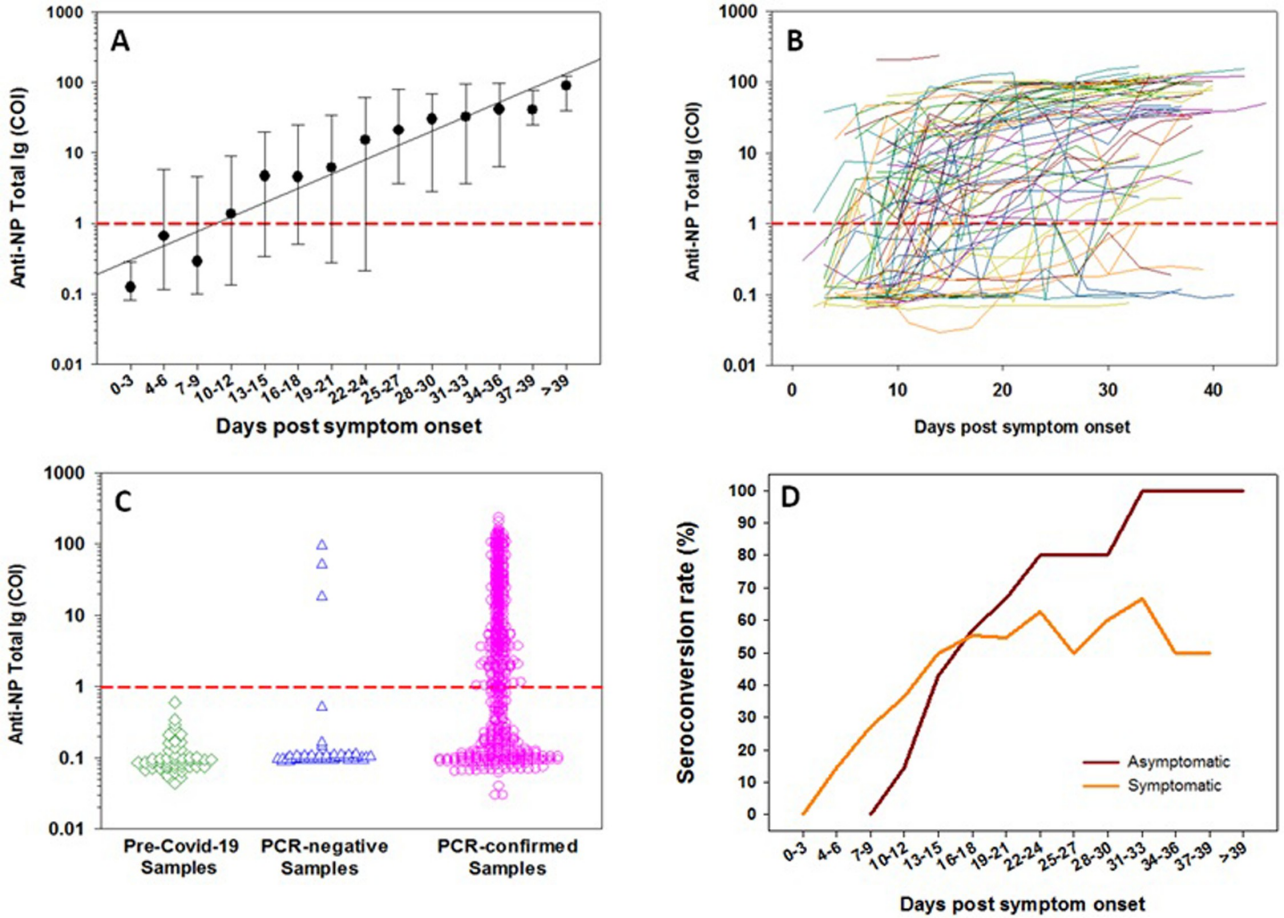
Characteristics of anti-NP total Ig response. Kinetics of median (IQR) anti-NP total Ig titers (as assessed by COI) at different time points after onset of symptoms (**A**); Kinetics of individual patients at different time points after onset of symptoms (**B**); scatter-plot of individual COI results in pre-COVID-19, PCR-negative, and PCR-confirmed COVID-19 patients (**C**); and seroconversion rate of anti-NP total Ig at different time points after onset of symptoms stratified by COVID-19 symptoms (**D**). The red horizontal line represents the cut-off value of the limit of detection of the Roche elecsys assay.

**Fig 3:**
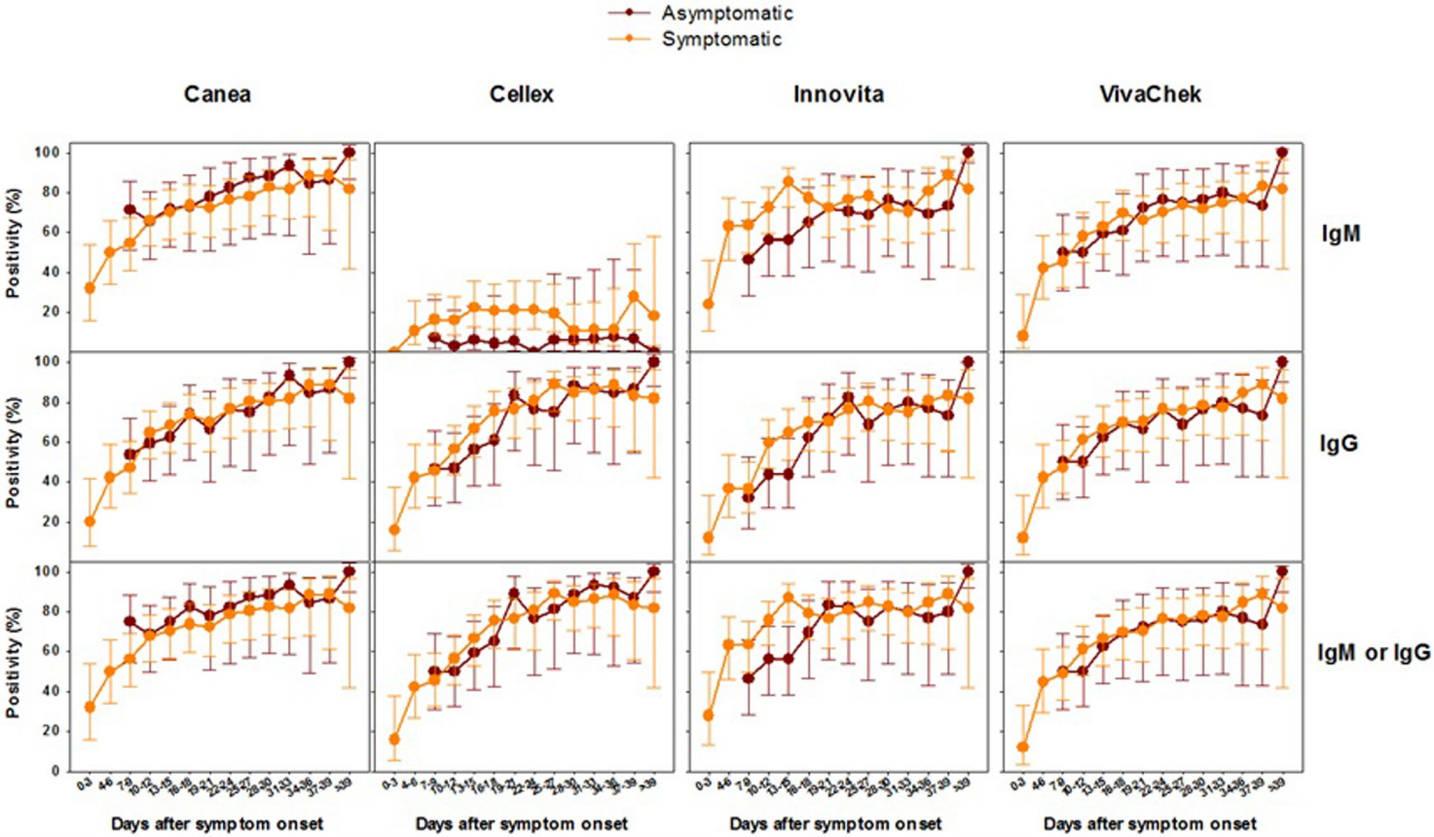
Kinetics of positivity rate (95% CI) of IgM, IgG, and IgM or IgG LFIAs at different time points stratified by COVID-19 symptoms.

The kinetics profile of antibody response is summarized in **Figs 1–3**. Serology testing was determined longitudinally in 102 patients, who provided a total of 802 plasma specimens (median of 8 paired assays per patient) for each of the LFIAs. For the Roche elecsys assay, only 631 specimens were evaluated due to insufficient reagent. The seropositivity rate of all assays evaluated was low at the time around admission to the hospital. Positivity rate was 32.0%, 16.0%, 28.0%, 12.0%, and 12.5%, for IgM or IgG by Canea, Cellex, Innovita, VivaChek, and Roche total Ig, respectively. However, during the first 15 days after the onset of symptoms, the positivity rate increased significantly reaching between 64.0% and 75.6% for IgM or IgG for the LFIAs, and 64.2% for Roche assay. Nonetheless, the positivity rate for Cellex IgM remained relatively low (16.3%) during the first 15 days after the onset of symptoms, and remained so during the whole period of hospitalization. Positive antibody titer was detectable (i.e. COI ≥ 1.0) as early as 10 to 12 days after onset of symptoms for the Roche elecsys total Ig assay (**Fig 2A**). During the period 2- to 3-weeks after the onset of symptoms, the positivity rate of all the assays was increased, ranging between 67.3% and 81.3%. Between 3 and 6 weeks after the onset of symptoms, the positivity rate continued to increase for all the assays, ranging from 84.6% to 90.9%. The positivity rate for IgM, IgG as well as combined IgM or IgG was not different for all of the assays assessed. Overall, longitudinal analysis revealed a heterogenous antibody responses profile to all assays tested (**Figs 1 and 2B and S2 Table**). In addition, we noted a trend towards a higher positivity rate of almost all assays among patients with symptomatic vs. asymptomatic (**Fig 3**), or severe COVID-19 compared to those with non-severe clinical presentation during the early phase of the infection (**S1 Fig**). However, the differences were not statistically significant. Notably, very few of the asymptomatic SARS-CoV-2 patients were positive to IgM by Cellex immunoassay (**Fig 3**).

**Fig 4:**
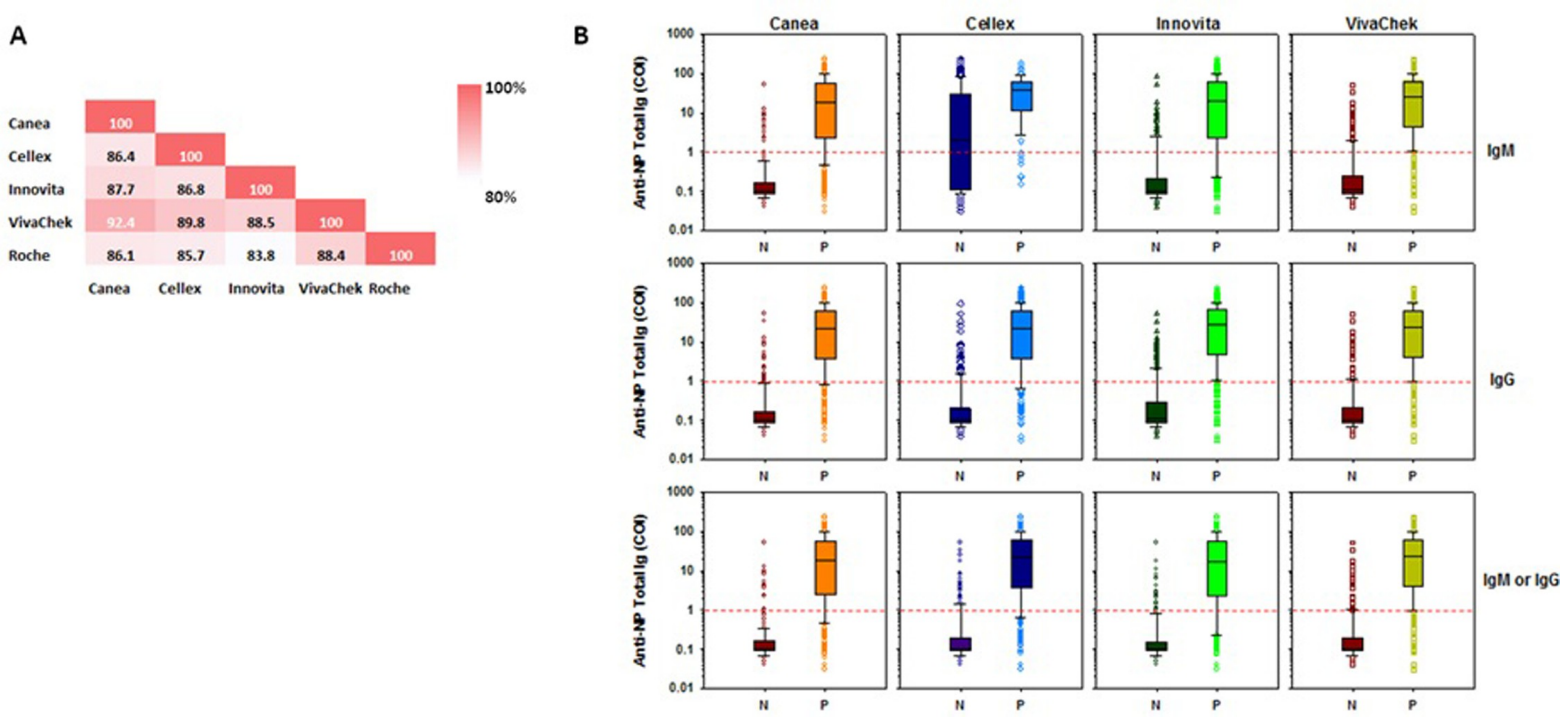
Agreement between serological assays for SARS-CoV-2. **A.** Percent agreement plotted across all assay combinations. Samples were designated as positive if IgM or IgG or total Ig was detected for each assay. **B.** IgM, IgG, and IgM or IgG LFIA results compared to anti-nucleocapsid protein total Ig titer (as determined by COI) of the Roche elecsys assay for all samples. The red horizontal line represents the cut-off value of the limit of detection of the Roche elecsys assay.

The specificity of the assays was evaluated using samples from RT-PCR negative individuals (n = 100) derived during the same enrollment period of the COVID-19 patients. In addition, we included pre-COVID-19 samples (n = 50). The results are summarized in **Table 2**. The specificity of the LFIAs ranged from 90.0% (95% CI: 82.2–94.59) to 99.0% (95% CI: 93.03–99.86) for IgM, from 92.0% (95% CI: 84.65–96.00) to 96.0% (95CI: 89.66–98.52) for IgG, and from 87.0% (95% CI: 78.72–92.37) to 96.0% (95% CI: 89.66–98.52) for IgM or IgG antibodies in RT-PCR negative specimens that were drawn during COVID-19 pandemic. In addition, specificity of the LFIAs ranged from 96.0% (95% CI: 84.72–99.05) to 100.0% (95CI: 92.75–100.00) for IgM, 100.0% (95CI: 92.75–100.00) for IgG, and from 96.0% (95% CI: 84.72–99.05) to 100.0% (95CI: 92.75–100.00) for IgM or IgG antibodies for samples collected during pre–pandemic period. For the Roche elecsys total Ig assay, the specificity was 94% (95% CI: 82.39–98.13) and 100.0% (95% CI: 92.75–100.00), for RT-PCR negative, and pre-COVID specimens, respectively (**Table 2 and Fig 2C**). Overall, 17/150 (11.3%) RT-PCR negative samples were found to be reactive in either one or more of the assays tested. Whereas 9 specimens reacted with only one assay, 6 were reactive with 4 different assays, 1 with 2 different assays, and 1 was reactive with 3 different assays. Only 2 samples appeared positive from those samples collected during the pre-COVID-19 pandemic period. The agreement among the different serology assays evaluated, and considering the results of combined IgM and IgG, was very good, ranging between 83.8% and 92.4% (**Fig 4A**). In addition, there was a direct correlation between the LFIAs test results and the Roche’s elecsys anti-NP total Ig titers (**Fig 4B**).

**Table 2:** Specificity in SARS-CoV-2RT-PCR negative collected during COVID-19 or pre–COVID-19 period.

SARS-CoV-2 RT-PCR negative samples												
Assay	IgM			IgG			IgM or IgG			Total Ig		
	No. specimens tested	No. positive	Specificity% (95% CI)	No. specimens tested	No. positive	Specificity% (95% CI)	No. specimens tested	No. positive	Specificity% (95% CI)	No. specimens tested	No. positive	Specificity% (95% CI)
**LFIAs**										–	–	–
Canea	100	8	92.0 (84.65–96.00)	100	8	92.0 (84.65–96.00)	100	8	92.0 (84.65–96.00)			
Cellex	100	1	99.0 (93.03–99.86)	100	4	96.0 (89.66–98.52)	100	4	96.0 (89.66–98.52)	–	–	–
Innovita	100	10	90.0 (82.24–94.59)	100	4	96.0 (89.66–98.52)	100	13	87.0 (78.72–92.37)	–	–	–
VivaChek	100	7	93.0 (85.88–96.67)	100	7	93.0 (85.88–96.67)	100	8	92.0 (84.65–96.00)	–	–	–
**ECLIA**										–	–	–
Roche	–	–	–	–	–	–	–	–	–	50	3	94.0 (82.39–98.13)
**Pre–COVID-19 pandemic samples**												
**LFIAs**										–	–	–
Canea	50	0	100.0 (92.75–100.0)	50	0	100.0 (92.75–100.0)	50	0	100.0 (92.75–100.0)	–	–	–
Cellex	50	0	100.0 (92.75–100.0)	50	0	100.0 (92.75–100.0)	50	0	100.0 (92.75–100.0)			
Innovita	50	2	96.0 (84.72–99.05)	50	0	100.0 (92.75–100.0)	50	2	96.0 (86.72–99.05)	–	–	–
VivaChek	50	0	100.0 (92.75–100.0)	50	0	100.0 (92.75–100.0)	50	0	100.0 (92.75–100.0)			
**ECLIA**										–	–	–
Roche	–	–	–	–	–	–	–	–	–	50	0	100.0 (92.75–100.0)

ECLIA: electro chemiluminescent immune assay; Ig: immunoglobulin; LFIAs: lateral flow immunoassays

Given that PPVs and NPVs are influenced by population prevalence of SARS-CoV-2 infection in a given time [20], we estimated the predictive values of five different assays, taking into consideration of population seroprevalences of 5%, 25% and 50% SARS-CoV-2infection (**Table 3**). Overall, the estimated PPV for IgM or IgG, or anti-NP total Ig in a scenario with low seroprevalence at 5% varies from 32.9% to 57.9%. Nonetheless, when the population seroprevalence increases to 25% and 50%, there is a corresponding increases in the estimated PPVs. The estimated NPV for IgM or IgG, or anti-NP total Ig in a low seroprevalence scenario (5%) is high, ranging between 98.7% and 99.1%. However, the estimated NPV in a high seroprevalence scenario (50%) is reduced significantly to 79.7% to 84.8%. Examples of a hypothetical scenario of the testing outcomes for 1000 people tested using the different assays we evaluated in a setting with a population seroprevalence of SARS-CoV-2 ranging from 5% to 50% is summarized in **S3 Table**. For example, in a population with a hypothetical seroprevalence of 5%, and using Cellex–an assay with a sensitivity of 83% would have 8 results per 1000 tested classified incorrectly as false negative. In addition, at a seroprevalence of 5%, this same assay with a specificity of 97% would have 28 out of 1000 tested classified incorrectly as false positive. However, at a seroprevalence of 50%, the same assay with the same sensitivity of 83% would provide 85 results for every 1000 cases tested classified incorrectly as false negative, and the same assay with the same specificity of 97% would result in only 15 cases for every 1000 tested that would be classified incorrectly as false positive. Overall, with increased population seroprevalence, PPV and false negative increase, and NPV and false positive decrease [20].

**Table 3:** Predictive values at different population seroprevalence rates.

Assay	Sensitivity (%)*	Specificity (%)**	Positive Predictive Value			Negative Predicitive Value		
			5%	25%	50%	5%	25%	50%
**IgM**								
Canea	80.4	94.7	44.2	83.4	93.8	99.0	93.5	82.5
Cellex	14.1	99.3	52.7	87.6	95.5	95.7	77.6	53.6
Innovita	74.7	93.3	37.1	78.9	91.8	98.6	91.7	78.7
VivaChek	72.8	95.3	45.1	83.9	93.4	98.5	91.0	77.8
**IgG**								
Canea	78.9	94.7	43.9	83.1	93.7	98.8	93.1	81.8
Cellex	81.7	96.7	56.3	89.1	96.1	99.0	94.1	84.0
Innovita	75.3	97.3	59.8	90.4	96.6	98.7	92.2	79.8
VivaChek	75.5	95.3	46.0	84.4	94.2	98.7	92.1	79.6
**IgM or IgG**								
Canea	81.2	94.7	44.5	83.5	93.8	99.0	93.8	83.5
Cellex	82.7	97.3	56.6	89.2	96.1	99.1	94.4	84.8
Innovita	80.6	90.0	32.9	75.6	90.3	98.9	93.4	82.5
VivaChek	76.0	94.7	42.8	82.6	93.4	98.7	92.2	79.7
**Total Ig**								
Roche elecsys	79.2	97.0	57.9	89.7	96.3	98.9	93.3	82.4

*Sensitivity calculated at after 15 days after symptom onset.

**Specificity calculated for all samples (both RT-PCR negative and pre-COVID specimens).

### Seroconversion of SARS-CoV-2 antibody

Patients who were antibody–negative at time of hospital admission, but who became antibody–positive during subsequent follow–up were considered as sero-converters. Overall, a total of 28 (27.5%) sero-converters were identified, 64 (62.8%) had already a positive antibody test result at time of admission, and 10 (9.8%) failed to mount a detectable antibody response by any of the assays tested throughout the course of follow-up.

**Fig 5:**
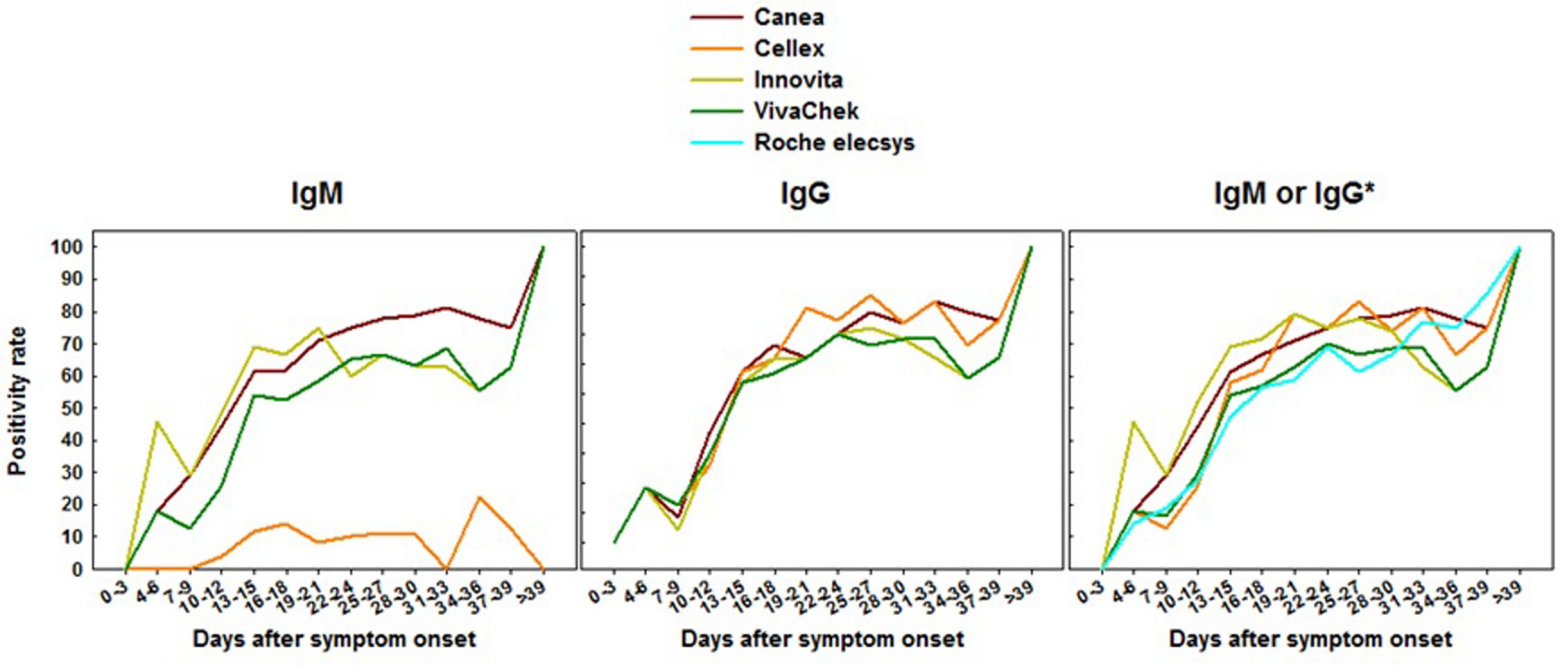
Kinetics of seroconversion rate of IgM, IgG, IgM or IgG LFIAs, and anti-NP total Ig at different time points after onset of symptoms. *Total Ig for Roche elecsys assay.

Seroconversion pattern as determined by different assays using various antibody isotypes are shown in **Fig 5**. Patients started to seroconvert as early as 4 to 6 days after onset of symptoms, reaching between 18.2% and 45.5% for the LIFAs IgM or IgG, and 14.3% for the Roche elecsys total Ig assay. The rate of seroconversion within the first 15 days after the onset of symptoms increased to 53.8% and 69.2% for the LFIAs IgM or IgG, and to 47.1% for the Roche elecsys assay. In addition, the seroconversion rate of all assays increased further during 2- to 3-weeks after onset of symptoms, ranging between 58.8% and 79.2%. The seroconversion rate continued to increase for all the assays, reaching 100% after 5.5 weeks after onset of symptoms. Nonetheless, seroconversion rate for Cellex IgM remained low throughout the course of follow-up. Overall, the median time for seroconversion was 11 (IQR: 9–15) days post symptom onset. The median time of seroconversion in the patients with severe COVID-19 tended to be shorter [10 (IQR: 8–11) days] when compared to non-severe COVID-19 patients [13 (IQR: 9–16) days]. However, this difference was not statistically significant (p = 0.251). Of the 28 patients who seroconverted, 11 (39.3%) were asymptomatic and 17 (60.7%) symptomatic. When stratified by symptoms, the median seroconversion time among symptomatic cases tended to be significantly shorter compared to asymptomatic patients [9 (IQR: 6–11) vs. 15 (IQR: 13–21) days; p = 0.002] (**Fig 2D**).

Of the remaining 74 COVID-19 patients included in the cohort, 10 (9.8%) had no detectable antibody response by each of the assay tested. These patients who failed to seroconvert included 5 mild/moderate, and 5 severe cases, with a median of 28 (IQR: 16–33) days follow-up after symptom onset (detailed information available in **S4 Table**).

## Discussion

Evaluation of serological tests for SARS-CoV-2 infection has already been described by several reports from HICs [7–13]. Nonetheless, reports on the evaluation of serology tests on specimens derived from longitudinally enrolled patients from LMICs is scarce [14]. Such evaluations are important since blood samples from LMICs are markedly different from HICs with different immune activation status, genetic backgrounds, and pathogen diversity, and load as compared to HICs. In this work, we assessed serology assays using specimens obtained in a LMIC setting (Ethiopia) from consecutively enrolled COVID–1 9 patients. Thus, we were able to determine the kinetics profile of antibody responses of individual patients, as well as seroconversion patterns. In addition, we were able to determine the performance of different serological assays in LMIC context.

The findings revealed that antibody responses were highly heterogeneous between individuals and assays, consistent with previous reports [21–28]. Comparable trends in antibody response kinetics observed between the different assays suggest that the different antigen targets (NP, N and S) were similarly immunogenic. Longitudinal kinetics assessment of the various serological assays revealed that positivity rate increased with increasing time after the onset of symptoms. Around 80% positivity rate was reached after 3 weeks of symptom onset. This finding concurs with the only other report existing, from Cameroon [14]. A positivity rate that is sufficient to identify all COVID-19 patients may require longer period of time after the onset of disease. This suggests that the mainstay of diagnosis of COVID-19 remains the detection of virus or virus products [2, 3]. There are conflicting reports regarding differences in positivity rate between non-severe and severe COVID-19 cases. Whereas some reports revealed that positivity rate among those with severe COVID-19 disease is significantly higher compared to those with non-severe disease [11, 12], others have demonstrated that there is no significant difference between the two groups [26]. Interestingly, it was shown that COVID-19 patients with more severe clinical presentation indeed exhibit a delayed production of antibodies compared to non-severe cases [22, 27]. In the current study, though there was a tendency towards increased positivity rate among severe compared to non-severe cases, we did not observe a significantly different pattern. In a previous report, we noted that being male is associated with COVID-19 severity [17]. However, in the current study, we did not observe differences in COVID-19 severity neither response to SARS-CoV-2 by gender, probably due to the smaller sample size. Though typically IgM appears earlier than IgG in other infectious events, this pattern is not typical in SARS-CoV-2 infection, as shown in the current study, as well as by previous investigators [26, 29], perhaps due to the fact that SARS-CoV-2 targets the lung epithelium initially. Furthermore, positivity rate for the assay Cellex IgM was found to be very low in this study, which is similar to previous report [30]. In contrast, others have reported higher positivity rate, ranging between 55.4% and 87.5% [22, 31]. The differences between the studies might be attributed to differences in sampling time, clinical severity status and study population immune responses.

The findings of this study revealed specificity of various serological assays tested > 90% for LFIAs and 97% for Roche elecsys. The findings that samples were reactive in particular to more than one assay may indicate the presence of cross-reacting antibodies, as has been reported in other African countries [15, 16]. Moreover, the possibility of false-negative RT-PCR test result should be considered, in particular for those samples collected during the COVID-19 pandemic. This notion is strengthened by the fact that in our cohort 16/17 samples that become reactive to serology were collected during the COVID-19 pandemic period. Thus, future in-depth evaluation of serological assays needs to consider such possibilities.

The positivity rate among the different assays that we tested was variable. The estimated PPV in a population with a seroprevalence of 5% was between 33% and 58%. Hence, an assay with high specificity is required to ascertain an acceptable PPV in a population with low seroprevalence [20]. Nonetheless, the estimated PPV increases significantly to over 90% in a population with seroprevalence of 50%. In such scenario, an assay that is needed is the one with a high sensitivity [20]. Overall, estimated PPV and NPV of each of the assay in a setting with a diverse population seroprevalence scenarios would certainly impact on the diagnostic testing outcomes. This needs to be considered during serosurveys [20].

Several reports have documented that the median time for IgM and IgG seroconversion is 7–14 days after the onset of symptoms [7, 10–12]. This is consistent with our finding showing a median seroconversion time of 11 days after onset of symptoms. Overall, these findings reflect the lower sensitivity of serological assays during the acute phase of the disease. In the current study, though there was a tendency towards earlier seroconversion rate among severe compared to non-severe cases, we did not observe a significantly different pattern. Nonetheless, when stratified by symptoms, those with a symptomatic clinical presentation seroconverted earlier when compared with those with asymptomatic presentation. Notably, we identified a total of 10 (9.8%) RT-PCR confirmed COVID-19 patients from our cohort who failed to seroconvert by any of the assays we tested. All these patients were symptomatic, and it is unlikely that these patients may have false-positive RT-PCR results. Except one case with HIV-1, none of the patients had immunosuppression. The number of days after onset of symptoms in two cases was < 5 days, and these patients might have seroconverted later. Our findings are somewhat higher compared to a previous report that showed that approximately 5% of symptomatic PCR-positive patients remain antibody negative [27], but lower than the one reported by Long et al showing lack of seroconversion in > 20% of cases [7]. The fact that some patients fail to mount an adequate level of antibody response indicates that certain serology tests may have suboptimal sensitivity in assessing past infections.

Strengths of the current study include evaluation of longitudinal serial samples from same individuals allowing us to determine the profile of antibody kinetics on individual patients, including exact seroconversion dates in some. In addition, a strength was the inclusion of asymptomatic cases, the analysis by COVID-19 disease severity, and the strict definition of controls (RT-PCR negative specimens obtained during the same period as well as samples collected before COVID-19 pandemic). Limitations associated with the study were the relatively small sample size, lack of evaluation of the neutralizing potential of the anti–SARS-CoV-2 antibodies, difficulty in selection of kits for evaluation, and the use of plasma rather than whole blood specimens to evaluate applicability of LFIAs as PoC tests for field use. In addition, we did not determine long-term outcome of SARS-CoV-2 antibody response several months after the onset of symptoms in the current study. In addition, antibody titer in this study was determined using elecysys total Ig platform that may have precluded the specific determination of antibody response against the spike protein of SARS-CoV-2.

SARS-CoV-2 seroprevalence studies done in African countries with commercial tests validated in Europe, the USA, or Asia is faced with several challenges [32]. Therefore, studies such as the one here contribute to the understanding of the characteristics and pattern of antibody response of COVID-19 patients in the African context. To the best of our knowledge, this study is the first prospective study to evaluate the kinetics of anti–SARS-CoV-2 antibody response evaluating seroconversion pattern among SARS-CoV-2 infected patients from Africa. The study underscores the need for a comprehensive evaluation of commercial assay before use for surveillance or in aiding the diagnostic work up of COVID-19 cases. In addition, our results, together with previous evidence conducted in Africa [33], suggest that combining different serology tests with antigen test may have a role in aiding the diagnosis of COVID-19 in resource-constrained settings. Furthermore, factors associated with failure to mount adequate SARS-CoV-2 antibody response need further research.

### Institutional review board statement

The study was reviewed and approved by the Health Research Ethics Review Committee of Mekelle University College of Health Sciences (#ERC 1769/2020).

### Informed consent statement

Written informed consent was obtained from all study participants. However, specimens used as controls from pre–pandemic period were specimens from biobank and individual study participant consent was not obtained. Nonetheless, all personal identifiers were de–linked from the original sources.

## Supporting information

S1 Fig Kinetics of antibody response among COVID-19 patients stratified by disease severity. (DOCX)

S1 Table Commercial kits used in the study and their characteristics. (DOCX)

S2 Table Raw data of positivity rate of SARS-CoV-2 antibodies in COVID-19 patients. (DOCX)

S3 Table Summary of hypothetical testing outcomes at various population SARS-CoV-2 seroprevalences. (DOCX)

S4 Table Characteristics of RT-PCR confirmed COVID-19 patients who failed to seroconvert. (DOCX)

## References

[1] Coronavirus COVID-19 global cases by the Center for Systems Science and Engineering (CSSE) at Johns Hopkins University. 2021, Available at: https://coronavirus.jhu.edu/map.html

[2] Bohn MK, Lippi G, Horvath A, Sethi S, Koch D, Ferrari M, et al. Molecular, serological, and biochemical diagnosis and monitoring of COVID-19: IFCC taskforce evaluation of the latest evidence. Clin Chem Lab Med 2020;58:1037–52 doi: 10.1515/cclm-2020-0722 32459192

[3] Sethuraman N, Jeremiah SS, Ryo A. Interpreting diagnostic tests for SARS-CoV-2. JAMA 2020; 323:2249–2251. doi: 10.1001/jama.2020.8259 32374370

[4] Gudbjartsson DF, Norddahl GL, Melsted P, Gunnarsdottir K, Holm H, Eythorsson E, et al. Humoral immune response to SARS-CoV-2 in Iceland. N Engl J Med 2020; 383:1724–1734. doi: 10.1056/NEJMoa2026116 32871063 PMC7494247

[5] Pollan M, Perez-Gomez B, Pastor-Barriuso R, Oteo J, Hernán MA, Pérez-Olmeda M, et al. Prevalence of SARS -CoV -2 in Spain (ENE -COVID): a nationwide, population -based seroepidemiological study. Lancet 2020; 396:535–544. doi: 10.1016/S0140-6736(20)31483-5 32645347 PMC7336131

[6] Wajnberg A, Mansour M, Leven E, Bouvier NM, Patel G, Firpo-Betancourt A, et al. Humoral response and PCR positivity in patients with COVID -19 in the New York City region, USA: an observational study. Lancet Microbe 2020; 1: e283–e289. doi: 10.1016/S2666-5247(20)30120-8 33015652 PMC7518831

[7] Long QX, Liu BZ, Deng HJ, Wu GC, Deng K, Chen YK, et al. Antibody responses to SARS-CoV-2 in patients with COVID-19. Nat Med 2020; 26:845–848. doi: 10.1038/s41591-020-0897-1 32350462

[8] Okba NMA, Müller MA, Li W, Wang C, GeurtsvanKessel CH, Corman VM, et al. Severe acute respiratory syndrome coronavirus 2-specific antibody responses in coronavirus disease 2019 patients. Emerg Infect Dis 2020; 26: 1478–1488. doi: 10.3201/eid2607.200841 32267220 PMC7323511

[9] Zhao J, Yuan Q, Wang H, Liu W, Liao X, Su Y, et al. Antibody responses to SARS-CoV-2 in patients of novel coronavirus disease 2019. Clin Infect Dis 2020; 71: 2027–2034. doi: 10.1093/cid/ciaa344 32221519 PMC7184337

[10] Yongchen Z, Shen H, Wang X, Shi X, Li Y, Yan J, et al. Different longitudinal patterns of nucleic acid and serology testing results based on disease severity of COVID-19 patients. Emerg Microbes Infect. 2020: 9: 833–836. doi: 10.1080/22221751.2020.1756699 32306864 PMC7241531

[11] Rijkers G, Murk JL, Wintermans B, van Looy B, van den Berge M, Veenemans J, et al. Differences in antibody kinetics and functionality between severe and mild severe acute respiratory syndrome coronavirus 2 infections. J Infect Dis 2020; 222:1265–1269. doi: 10.1093/infdis/jiaa463 32726417 PMC7454692

[12] Margolin E, Burgers WA, Sturrock ED, Mendelson M, Chapman R, Douglass N, et al. Prospects for SARS-CoV-2 diagnostics, therapeutics and vaccines in Africa. Nat Rev Microbiol 2020; 18: 690–704. doi: 10.1038/s41579-020-00441-3 32913297 PMC7481764

[13] Lisboa Bastos M, Tavaziva G, Abidi SK, Campbell JR, Haraoui LP, Johnston JC, et al. Diagnostic accuracy of serological tests for covid-19: systematic review and meta-analysis. BMJ 2020; 370: m2516. doi: 10.1136/bmj.m2516 32611558 PMC7327913

[14] Fai KN, Corine TM, Bebell LM, Mboringong AB, Nguimbis EBPT, Nsaibirni R, et al. Serological response to SARS-CoV-2 in an African population. Sci African 2021; 12: e00802. 10.1016/j.sciaf.2021.e00802 PMC8164732 34095639

[15] Tso FY, Lidenge SJ, Peñaa PB, Clegg AA, Ngowi JR, Mwaiselage J, et al. High prevalence of pre-existing serological cross-reactivity against severe acute respiratory syndrome coronavirus-2 (SARSCoV-2) in sub-Saharan Africa. Int J Infect Dis 2021; 102: 577–583. doi: 10.1016/j.ijid.2020.10.104 33176202 PMC7648883

[16] Yadouleton A, Sander AL, Moreira-Soto A, Tchibozo C, Hounkanrin G, Badou Y, et al. Limited specificity of serologic tests for SARS-CoV-2 antibody detection, Benin. Emerg Infec Dis 2021; 27: 233–237. doi: 10.3201/eid2701.203281 33261717 PMC7774555

[17] Abraha HE, Gessesse Z, Gebrecherkos T, Kebede Y, Weldegiargis AW, Tequare MH, et al. Clinical features and risk factors associated with morbidity and mortality among COVID-19 patients in Northern Ethiopia. Int J Infect Dis. (2021) 105: 776–783. doi: 10.1016/j.ijid.2021.03.037 33741488 PMC7962557

[18] International Severe Acute Respiratory and Emerging Infection Consortium (ISARIC). COVID-19 CRF. https://isaric.tghn.org/COVID-19- CRF/. (Accessed Aug 28, 2020).

[19] World Health Organization. Report of the WHO-China Joint Mission on Coronavirus Disease 2019 (COVID-19): WHO, Geneva, Switzerland. https://www.who.int/docs/default-source/coronaviruse/who-china-jointmission-on-covid-19-final-report.pdf. (Accessed 01 July 2020).

[20] Peeling RW, Olliaro PL. The time to do serosurveys for COVID-19 is now. Lancet Resp Med 2020; 8: 836–838. 10.1016/S2213-2600(20)30313-1 PMC7380934 32717209

[21] Whitman JD, Hiatt J, Mowery CT, Shy BR, Yu R, Yamamoto TN, et al. Evaluation of SARS-CoV-2 serology assays reveals a range of test performance. Nat Biotechnol 2020; 38: 1174–1183. doi: 10.1038/s41587-020-0659-0 32855547 PMC7740072

[22] Serre-Miranda C, Nobrega C, Roque S, Canto-Gomes J, Silva CS, Vieira N, et al. Performance assessment of 11 commercial serological tests for SARS-CoV-2 on hospitalised COVID-19 patients. Int J Infect Dis 2021; 104: 661–669. doi: 10.1016/j.ijid.2021.01.038 33484862 PMC7817432

[23] Charlton CL, Kanji JN, Johal K, Bailey A, Plitt SS, MacDonald C, et al. Evaluation of six commercial mid to high volume antibody and six point of care lateral flow assays for detection of SARS-CoV-2 antibodies. J Clin Microbiol 2020; 58: e01361–20. doi: 10.1128/JCM.01361-20 32665420 PMC7512179

[24] Therrien C, Serhir B, Bélanger-Collard M, Skrzypczak J, Shank DK, Renaud C, et al. Multicenter evaluation of the clinical performance and the neutralizing antibody activity prediction properties of 10 high-throughput serological assays used in clinical laboratories. J Clin Microbiol 2021; 59:e02511–e02520. doi: 10.1128/JCM.02511-20 33303562 PMC8106733

[25] Chen SY, Lee YL, Lin YC, Lee NY, Liao CH, Hung YP, et al. Multicenter evaluation of two chemiluminescence and three lateral flow immunoassays for the diagnosis of COVID-19 and assessment of antibody dynamic responses to SARS-CoV-2 in Taiwan. Emer Micro Infect, 2020; 9: 2157–2168 doi: 10.1080/22221751.2020.1825016 32940547 PMC7580576

[26] van Elslande J, Houben E, Depypere M, Brackenier A, Desmet S, André E, et al. Diagnostic performance of seven rapid IgG/IgM antibody tests and the Euroimmun IgA/IgG ELISA in COVID-19 patients. Clin Microbiol Infect 2020; 26: 1082–1087. doi: 10.1016/j.cmi.2020.05.023 32473953 PMC7255746

[27] Oved K, Olmer L, Shemer-Avni Y, Wolf T, Supino-Rosin L, Prajgrod G, et al. Multi-center nationwide comparison of seven serology assays reveals a SARS-CoV-2 non-responding seronegative subpopulation. EClinicalMedicine 2020; 29: 100651. doi: 10.1016/j.eclinm.2020.100651 33235985 PMC7676374

[28] Basgalupp S, dos Santos G, Bessel M, Garcia L, de Moura AC, Rocha AC, et al. Diagnostic properties of three SARS-CoV-2 antibody tests. Diagnostics 2021; 11: 1441–1152. doi: 10.3390/diagnostics11081441 34441375 PMC8393643

[29] Ma H, Zeng W, He H, Zhao D, Jiang D, Zhou P, et al. Serum IgA, IgM, and IgG responses in COVID-19. Cell Mol Immunol. 2020; 17:773–775. doi: 10.1038/s41423-020-0474-z 32467617 PMC7331804

[30] Ong DSY, de Man SJ, Lindeboom FA, Koeleman JGM. Comparison of diagnostic accuracies of rapid serological tests and ELISA to molecular diagnostics in patients with suspected coronavirus disease 2019 presenting to the hospital. Clin Microbiol Infect 2020; 26:1094.e7-1094.e. doi: 10.1016/j.cmi.2020.05.028 32502646 PMC7265854

[31] Trombetta BA, Kandigian SE, Kitchen RR, Grauwet K, Webb PK, Miller GA, et al. Evaluation of serological lateral flow assays for severe acute respiratory syndrome coronavirus-2. BMC Infect Dis 2021; 21:580–594. doi: 10.1186/s12879-021-06257-7 34134647 PMC8206878

[32] Nkuba Ndaye A, Hoxha A, Madinga J, Mariën J, Peeters M, Leendertz FH, et al. Challenges in interpreting SARS-CoV-2 serological results in African countries. Lancet Glob Health 2021; 9: e588–e589. doi: 10.1016/S2214-109X(21)00060-7 33609481 PMC7906714

[33] Boum Y, Fai KN, Nicolay B, Mboringong AB, Bebell LM, Ndifon M, et al. Performance and operational feasibility of antigen and antibody rapid diagnostic tests for COVID-19 in symptomatic and asymptomatic patients in Cameroon: a clinical, prospective, diagnostic accuracy study. Lancet Infect Dis 2021; 21: 1089–1096. doi: 10.1016/S1473-3099(21)00132-8 33773618 PMC7993929

