# Supplementary figures and images for: Longitudinal profile of antibody response to SARS-CoV-2 in patients with COVID-19 in a setting from Sub–Saharan Africa: A prospective longitudinal study
Source: PLoS One. 2022 Mar 23;17(3):e0263627. doi: 10.1371/journal.pone.0263627 (PMC8942258; doi:10.1371/journal.pone.0263627)

**S1 Fig. Kinetics of antibody response among COVID-19 patients stratified by disease severity.**


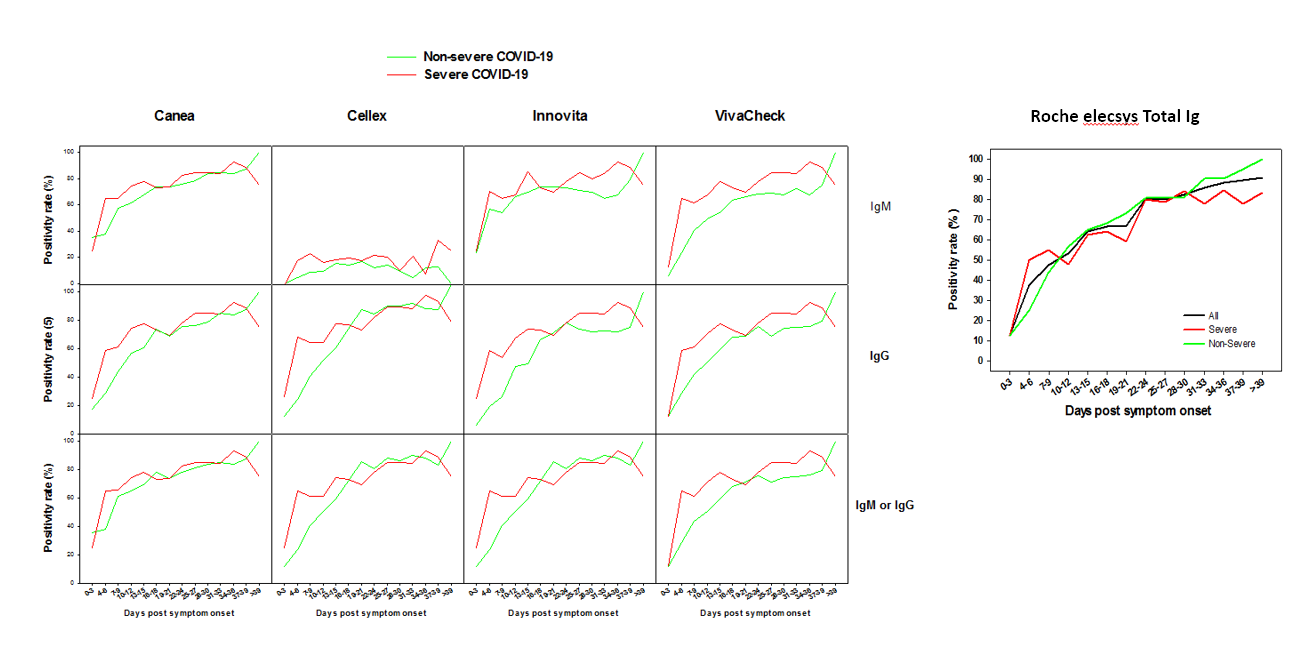

Supplement: S1 Fig — (DOCX) [file pone.0263627.s001.docx]
