# Supplementary material for: Longitudinal profile of antibody response to SARS-CoV-2 in patients with COVID-19 in a setting from Sub–Saharan Africa: A prospective longitudinal study
Source: PLoS One. 2022 Mar 23;17(3):e0263627. doi: 10.1371/journal.pone.0263627 (PMC8942258; doi:10.1371/journal.pone.0263627)
Supplement: S1 Table — (DOCX) [file pone.0263627.s002.docx]

**S1 Table. Commercial kits used in the study and their characteristics.**

| **Kit name** | **Assay**  **type** | **SARS-CoV-2 antigen target** | **Anti-SARS-CoV-2 Antibodies detected** | **Manufacturer’s reported** | |
| --- | --- | --- | --- | --- | --- |
|  |  |  |  | **Sensitivity** | **Specificity** |
| Canea | LFIA | N and S | IgM and IgG | 91.8% | 99.2% |
| Cellex | LFIA | N and S | IgM and IgG | 93.8% | 96.0% |
| Innovita | LFIA | N and S | IgM and IgG | 100.0% | 97.5% |
| VivaCheck | LFIA | N and S | IgM and IgG | 97.1% | 100.0% |
| Roche elecsys | ECLIA | NP | Total antibodies | 100.0%* | 100.0% |

Abbreviations: ECLIA: electrochemiluminescent immunoassay; LFIA: lateral-flow immuno-chromatographic assay; N: nuclear; NP: nucleocapsid protein; S: spike

*For samples collected ≥ 15 days after symptom onset.
