# Supplementary material for: Longitudinal profile of antibody response to SARS-CoV-2 in patients with COVID-19 in a setting from Sub–Saharan Africa: A prospective longitudinal study
Source: PLoS One. 2022 Mar 23;17(3):e0263627. doi: 10.1371/journal.pone.0263627 (PMC8942258; doi:10.1371/journal.pone.0263627)
Supplement: S2 Table — (DOCX) [file pone.0263627.s003.docx]

**S2 Table. Raw data of positivity rate of SARS-CoV-2 antibodies in COVID-19 patients.**

| **Assay** | **No. specimens tested** | **IgM** | |  | **IgG** | |  | **IgM or IgG** | |
| --- | --- | --- | --- | --- | --- | --- | --- | --- | --- |
|  |  | **No. positive** | **%**  **(95% CI)** |  | **No. positive** | **%**  **(95% CI)** |  | **No. positive** | **%**  **(95% CI)** |
| **LFIAs** |  |  |  |  |  |  |  |  |  |
| Canea |  |  |  |  |  |  |  |  |  |
| 0–3 d | 25 | 8 | 32.0 (16.02-53.72) |  | 5 | 20.0 (8.02-41.75) |  | 8 | 32.0 (16.02-53.73) |
| 4–6 d | 38 | 19 | 50.0 (33.93-66.07) |  | 16 | 42.1 (27.03-58.81) |  | 19 | 50.0 (33.93-66.07) |
| 7–9 d | 83 | 50 | 60.2 (49.17-70.36) |  | 41 | 49.4 (38.62-60.24) |  | 52 | 62.7 (51.58-72.54) |
| 10–12 d | 94 | 62 | 66.0 (55.65-74.95) |  | 59 | 62.8 (52.40-72.07) |  | 64 | 68.1 (57.83-76.84) |
| 13–15 d | 86 | 61 | 70.9 (60.28-79.69) |  | 57 | 66.3 (55.47-75.62) |  | 62 | 72.1 (61.50-80.69) |
| 16–18 d | 76 | 56 | 73.7 (62.42-82.52) |  | 56 | 73.7 (62.42-82.52) |  | 58 | 76.3 (65.23-84.70) |
| 19–21 d | 65 | 48 | 73.8 (61.53-83.29) |  | 45 | 69.2 (56.70-79.45) |  | 48 | 73.8 (61.53-83.29) |
| 22–24 d | 64 | 50 | 78.1 (66.01-86.78) |  | 49 | 76.6 (64.32-85.55) |  | 51 | 79.7 (67.72-88.00) |
| 25–27 d | 62 | 50 | 80.7 (68.55-88.85) |  | 49 | 79.0 (66.71-87.61) |  | 51 | 82.3 (70.34-90.06) |
| 28–30 d | 63 | 53 | 84.1 (72.57-91.39) |  | 51 | 81.0 (69.01-89.03) |  | 53 | 84.1 (72.57-91.39) |
| 31–33 d | 59 | 50 | 84.8 (72.79-92.03) |  | 50 | 84.7 (72.79-92.03) |  | 50 | 84.7 (72.79-92.03) |
| 34–36 d | 39 | 34 | 87.2 (71.80-94.78) |  | 34 | 87.2 (71.80-94.78) |  | 34 | 87.2 (71.80-94.78) |
| 37–39 d | 33 | 29 | 87.9 (70.63-95.62) |  | 29 | 87.9 (70.63-95.62) |  | 29 | 87.9 (70.63-95.62) |
| ≥ 40 d | 13 | 11 | 84.6 (49.04-96.92) |  | 11 | 84.6 (49.04-96.92) |  | 11 | 84.6 (49.04-96.92) |
| Cellex |  |  |  |  |  |  |  |  |  |
| 0–3 d | 25 | 0 | 0.00 (..-..) |  | 4 | 16.0 (5.69-37.54) |  | 4 | 16.0 (5.69-37.54) |
| 4–6 d | 38 | 4 | 10.5 (3.82-25.83) |  | 16 | 42.1 (27.03-58.81) |  | 16 | 42.1 (27.03-58.81) |
| 7–9 d | 83 | 11 | 13.3 (7.40-22.60) |  | 38 | 45.8 (35.21-56.75) |  | 39 | 47.0 (36.34-57.92) |
| 10–12 d | 94 | 11 | 11.7 (6.53-20.10) |  | 50 | 53.2 (42.93-63.19) |  | 51 | 54.3 (43.96-64.20) |
| 13–15 d | 86 | 14 | 16.3 (9.78-25.86) |  | 54 | 62.8 (51.93-72.50) |  | 55 | 64.0 (53.10-73.55) |
| 16–18 d | 76 | 12 | 15.8 (9.07-26.05) |  | 54 | 71.1 (59.65-80.30) |  | 55 | 72.4 (61.03-81.42) |
| 19–21 d | 65 | 11 | 16.9 (9.47-28.39) |  | 51 | 78.5 (66.49-86.99) |  | 52 | 80.0 (68.18-88.19) |
| 22–24 d | 64 | 10 | 15.6 (8.47-27.03) |  | 51 | 79.7 (67.72-88.00) |  | 51 | 79.7 (67.72-88.00) |
| 25–27 d | 62 | 10 | 16.1 (8.75-27.84) |  | 53 | 85.5 (74.01-92.41) |  | 54 | 87.1 (75.87-93.54) |
| 28–30 d | 63 | 6 | 9.5 (4.24-20.00) |  | 54 | 85.7 (74.39-92.53) |  | 54 | 85.7 (74.39-92.53) |
| 31–33 d | 59 | 6 | 10.2 (4.53-21.27) |  | 51 | 86.4 (74.74-93.21) |  | 52 | 88.1 (76.72-94.36) |
| 34–36 d | 39 | 4 | 10.3 (3.73-25.23) |  | 34 | 87.2 (71.80-94.78) |  | 35 | 89.7 (74.77-96.27) |
| 37–39 d | 33 | 6 | 18.2 (8.03-36.11) |  | 28 | 84.8 (67.23-93.86) |  | 28 | 84.8 (67.23-93.86) |
| ≥ 40 d | 13 | 2 | 15.4 (3.08-50.96) |  | 11 | 84.6 (49.04-96.92) |  | 11 | 84.6 (49.04-96.92) |
| Innovita |  |  |  |  |  |  |  |  |  |
| 0–3 d | 25 | 6 | 24.0 (10.54-45.85) |  | 3 | 12.0 (3.60-33.27) |  | 7 | 28.0 (13.21-49.85) |
| 4–6 d | 38 | 24 | 63.2 (46.22-77.37) |  | 14 | 36.8 (46.22-77.37) |  | 24 | 63.2 (46.22-77.37) |
| 7–9 d | 83 | 48 | 57.8 (46.78-68.15) |  | 29 | 34.9 (25.30-45.99) |  | 48 | 57.8 (46.78-68.15) |
| 10–12 d | 94 | 63 | 67.0 (56.74-75.90) |  | 51 | 54.3 (43.96-64.19) |  | 65 | 69.2 (58.93-77.78) |
| 13–15 d | 86 | 64 | 74.4 (63.96-82.67) |  | 49 | 57.0 (46.14-67.18) |  | 65 | 75.6 (65.20-83.64) |
| 16–18 d | 76 | 56 | 73.7 (62.42-82.52) |  | 52 | 68.4 (56.91-78.04) |  | 58 | 76.3 (65.22-84.70) |
| 19–21 d | 65 | 47 | 72.3 (59.91-82.02) |  | 46 | 70.8 (58.30-80.74) |  | 51 | 78.5 (66.49-86.99) |
| 22–24 d | 64 | 48 | 75.0 (62.65-84.29) |  | 50 | 78.1 (66.01-86.78) |  | 52 | 81.3 (69.45-89.20) |
| 25–27 d | 62 | 47 | 75.8 (63.28-85.07) |  | 48 | 77.4 (65.02-86.35) |  | 51 | 82.3 (70.34-90.06) |
| 28–30 d | 63 | 46 | 73.0 (60.43-82.74) |  | 48 | 76.2 (63.81-85.31) |  | 52 | 82.5 (70.78-90.22) |
| 31–33 d | 59 | 42 | 71.2 (58.03-81.53) |  | 45 | 76.3 (63.41-85.64) |  | 47 | 79.7 (67.09-88.27) |
| 34–36 d | 39 | 30 | 76.9 (60.46-87.90) |  | 31 | 79.5 (63.21-89.73) |  | 32 | 82.1 (66.02-91.49) |
| 37–39 d | 33 | 27 | 81.8 (63.89-91.97) |  | 26 | 78.8 (60.62-89.96) |  | 28 | 84.9 (67.23-93.86) |
| ≥ 40 d | 13 | 11 | 84.6 (49.04-96.92) |  | 11 | 84.6 (49.04-96.92) |  | 11 | 84.6 (49.04-96.92) |
| VivaChek |  |  |  |  |  |  |  |  |  |
| 0–3 d | 25 | 2 | 8.0 (1.81-29.12) |  | 3 | 12.0 (3.60-33.27) |  | 3 | 12.0 (3.60-33.27) |
| 4–6 d | 38 | 16 | 42.1 (27.03-58.81) |  | 16 | 42.1 (27.03-58.81) |  | 17 | 44.7 (29.29-61.27) |
| 7–9 d | 83 | 39 | 47.0 (36.34-57.92) |  | 40 | 48.2 (37.47-59.08) |  | 41 | 49.4 (38.62-60.24) |
| 10–12 d | 94 | 52 | 55.3 (45.00-65.20) |  | 54 | 57.4 (47.09-67.19) |  | 54 | 57.5 (47.09-67.19) |
| 13–15 d | 86 | 53 | 61.6 (50.76-71.45) |  | 56 | 65.1 (54.28-74.59) |  | 56 | 65.1 (54.28-74.59) |
| 16–18 d | 76 | 51 | 67.1 (55.56-76.90) |  | 53 | 69.7 (58.28-79.18) |  | 53 | 69.7 (58.28-79.18) |
| 19–21 d | 65 | 44 | 67.7 (55.12-78.14) |  | 45 | 69.2 (56.71-79.45) |  | 46 | 70.8 (58.30-80.74) |
| 22–24 d | 64 | 46 | 71.9 (59.35-81.73) |  | 49 | 76.6 (64.32-85.55) |  | 49 | 76.6 (64.32-85.55) |
| 25–27 d | 62 | 46 | 74.2 (61.56-83.77) |  | 46 | 74.2 (61.56-83.77) |  | 47 | 75.8 (63.28-85.07) |
| 28–30 d | 63 | 46 | 73.0 (60.43-82.74) |  | 49 | 77.8 (65.52-86.57) |  | 49 | 77.8 (65.52-86.57) |
| 31–33 d | 59 | 45 | 76.3 (63.41-85.64) |  | 46 | 78.0 (65.24-86.96) |  | 46 | 78.0 (65.24-86.96) |
| 34–36 d | 39 | 30 | 76.9 (60.46-87.90) |  | 32 | 82.1 (66.02-91.49) |  | 32 | 82.1 (66.02-91.49) |
| 37–39 d | 33 | 26 | 78.8 (60.62-89.96) |  | 27 | 81.8 (63.89-91.97) |  | 27 | 81.8 (63.89-91.97) |
| ≥ 40 d | 13 | 11 | 84.6 (49.04-96.92) |  | 11 | 84.6 (49.04-96.92) |  | 11 | 84.6 (49.04-96.92) |
| **ECLIA** |  |  |  |  |  |  |  |  |  |
| Roche* |  |  |  |  |  |  |  |  |  |
| 0–3 d | 16 | .. | .. |  | .. | .. |  | 2 | 12.5 (2.63–43.00) |
| 4–6 d | 24 | .. | .. |  | .. | .. |  | 9 | 37.5 (19.75–59.39) |
| 7–9 d | 61 | .. | .. |  | .. | .. |  | 29 | 47.5 (35.08–60.32) |
| 10–12 d | 73 | .. | .. |  | .. | .. |  | 39 | 53.4 (41.73–64.75) |
| 13–15 d | 67 | .. | .. |  | .. | .. |  | 43 | 64.2 (51.76–74.95) |
| 16–18 d | 63 | .. | .. |  | .. | .. |  | 43 | 68.3 (55.48–78.77) |
| 19–21 d | 52 | .. | .. |  | .. | .. |  | 35 | 67.3 (53.07–78.94) |
| 22–24 d | 51 | .. | .. |  | .. | .. |  | 41 | 80.4 (66.72–89.34) |
| 25–27 d | 50 | .. | .. |  | .. | .. |  | 40 | 80.0 (66.12–89.13) |
| 28–30 d | 51 | .. | .. |  | .. | .. |  | 42 | 82.4 (68.90–90.77) |
| 31–33 d | 49 | .. | .. |  | .. | .. |  | 42 | 85.7 (72.36–93.22) |
| 34–36 d | 34 | .. | .. |  | .. | .. |  | 30 | 88.2 (71.42–95.75) |
| 37–39 d | 29 | .. | .. |  | .. | .. |  | 26 | 89.7 (70.86–96.87) |
| ≥ 40 d | 11 | .. | .. |  | .. | .. |  | 10 | 90.9 (46.30–99.15) |

ECLIA: electro chemiluminescent immune assay; LFIAs: lateral flow immunoassays

*For Roche, antibody measured is total Ig.
