# Supplementary material for: Longitudinal profile of antibody response to SARS-CoV-2 in patients with COVID-19 in a setting from Sub–Saharan Africa: A prospective longitudinal study
Source: PLoS One. 2022 Mar 23;17(3):e0263627. doi: 10.1371/journal.pone.0263627 (PMC8942258; doi:10.1371/journal.pone.0263627)
Supplement: S3 Table — (DOCX) [file pone.0263627.s004.docx]

**S3 Table. Summary of hypothetical testing outcomes at various population SARS-CoV-2 seroprevalences**

| **Population infected with SARS-COV-2** | **Se** | **Sp** | **Classification by serology test** | **Results per 1000 persons tested (95% CI)** | | | | | |
| --- | --- | --- | --- | --- | --- | --- | --- | --- | --- |
|  |  |  |  |  | **5% prevalence** |  | **25% prevalence** |  | **50% prevalence** |
| Canea IgM or IgG | 81 | - | Correctly classified as infected (TP) |  | 41 |  | 203 |  | 405 |
|  |  |  | Incorrectly classified as not infected (FN) |  | 9 |  | 48 |  | 95 |
| Cellex IgM or IgG | 83 | - | Correctly classified as infected (TP) |  | 42 |  | 208 |  | 415 |
|  |  |  | Incorrectly classified as not infected (FN) |  | 8 |  | 42 |  | 85 |
| Innovita IgM or IgG | 81 | - | Correctly classified as infected (TP) |  | 41 |  | 203 |  | 405 |
|  |  |  | Incorrectly classified as not infected (FN) |  | 9 |  | 47 |  | 95 |
| VivaChek IgM or IgG | 76 | - | Correctly classified as infected (TP) |  | 38 |  | 190 |  | 380 |
|  |  |  | Incorrectly classified as not infected (FN) |  | 12 |  | 60 |  | 120 |
| Roche elecsys total Ig | 79 | - | Correctly classified as infected (TP) |  | 40 |  | 198 |  | 395 |
|  |  |  | Incorrectly classified as not infected (FN) |  | 10 |  | 52 |  | 105 |
| **Population not infected with SARS-COV-2**** |  |  |  |  |  |  |  |  |  |
| Canea IgM or IgG | - | 95 | Correctly classified as not infected (TN) |  | 903 |  | 713 |  | 475 |
|  |  |  | Incorrectly classified as infected (FP) |  | 47 |  | 37 |  | 25 |
| Cellex IgM or IgG | - | 97 | Correctly classified as not infected (TN) |  | 922 |  | 728 |  | 485 |
|  |  |  | Incorrectly classified as infected (FP) |  | 28 |  | 22 |  | 15 |
| Innovita IgM or IgG | - | 90 | Correctly classified as not infected (TN) |  | 855 |  | 675 |  | 450 |
|  |  |  | Incorrectly classified as infected (FP) |  | 95 |  | 75 |  | 50 |
| VivaChek IgM or IgG | - | 95 | Correctly classified as not infected (TN) |  | 903 |  | 713 |  | 475 |
|  |  |  | Incorrectly classified as infected (FP) |  | 47 |  | 37 |  | 25 |
| Roche elecsys total Ig | - | 97 | Correctly classified as not infected (TN) |  | 922 |  | 728 |  | 485 |
|  |  |  | Incorrectly classified as infected (FP) |  | 28 |  | 22 |  | 15 |

*Se: sensitivity; Sp: specificity

**Enrolled during COVID-19 pandemic from individuals not suspected of SARS-CoV-2 infection as well as pre-COVID-19 period**.**
