# Supplementary material for: Longitudinal profile of antibody response to SARS-CoV-2 in patients with COVID-19 in a setting from Sub–Saharan Africa: A prospective longitudinal study
Source: PLoS One. 2022 Mar 23;17(3):e0263627. doi: 10.1371/journal.pone.0263627 (PMC8942258; doi:10.1371/journal.pone.0263627)
Supplement: S4 Table — (DOCX) [file pone.0263627.s005.docx]

**S4 Table. Characteristics of RT-PCR confirmed COVID-19 patients who failed to seroconvert.**

| **Pt. no.:** | **Sex** | **Age (years)** | **Follow-up days** | **Total # of samples tested** | **Covid-19 Severity** | **Co-morbidity** |
| --- | --- | --- | --- | --- | --- | --- |
| 1 | Male | 30 | 33 | 11 | Mild/moderate | None |
| 2 | Female | 24 | 3 | 2 | Mild/moderate | None |
| 3 | Male | 24 | 5 | 2 | Mild/moderate | None |
| 4 | Male | 32 | 31 | 11 | Mild/moderate | None |
| 5 | Female | 24 | 16 | 5 | Mild/moderate | None |
| 6 | Male | 34 | 40 | 6 | Severe | CVD |
| 7 | Female | 50 | 42 | 10 | Severe | CVD |
| 8 | Male | 30 | 24 | 6 | Severe | None |
| 9 | Male | 38 | 19 | 6 | Severe | HIV-1 |
| 10 | Male | 66 | 33 | 11 | Severe | None |

Abbreviations= CVD: cardiovascular disease; HIV-1: human immunodeficiency virus type-1
